# Trends in lung cancer incidence and mortality in Croatia, 1988 to 2008

**DOI:** 10.3325/cmj.2012.53.93

**Published:** 2012-04

**Authors:** Mateja Janković, Miroslav Samaržija, Marko Jakopović, Tomislav Kuliš, Ariana Znaor

**Affiliations:** 1 Department for Respiratory Diseases University Hospital Centre Zagreb, University of Zagreb School of Medicine, Zagreb, Croatia; 2 Department of Urology, University Hospital Centre Zagreb, University of Zagreb School of Medicine, Zagreb, Croatia; 3 Croatian National Cancer Registry, Croatian National Institute of Public Health, Zagreb, Croatia; 4 Andrija Štampar School of Public Health, University of Zagreb School of Medicine, Zagreb, Croatia

## Abstract

**Aim:**

To describe and interpret lung cancer incidence and mortality trends in Croatia between 1988 and 2008.

**Methods:**

Incidence data on lung cancer for the period 1988-2008 were obtained from the Croatian National Cancer Registry, while mortality data were obtained from the World Health Organization mortality database. Population estimates for Croatia were obtained from the Population Division of the Department of Economic and Social Affairs of the United Nations. We also calculated and analyzed age-standardized incidence and mortality rates. To describe time incidence and mortality trends, we used joinpoint regression analysis.

**Results:**

Lung cancer incidence and mortality rates in men decreased significantly in all age groups younger than 70 years. Age-standardized incidence rates in men decreased significantly by -1.3% annually. Joinpoint analysis of mortality in men identified three trends, and average annual percent change (AAPC) decreased significantly by -1.1%. Lung cancer incidence and mortality rates in women increased significantly in all age groups older than 40 years and decreased in younger women (30-39- years). Age-standardized incidence rates increased significantly by 1.7% annually. Joinpoint analysis of age-standardized mortality rates in women identified two trends, and AAPC increased significantly by 1.9%.

**Conclusion:**

Despite the overall decreasing trend, Croatia is still among the European countries with the highest male lung cancer incidence and mortality. Although the incidence trend in women is increasing, their age standardized incidence rates are still 5-fold lower than in men. These trends follow the observed decrease and increase in the prevalence of male and female smokers, respectively. These findings indicate the need for further introduction of smoking prevention and cessation policies targeting younger population, particularly women.

Lung cancer is the most common malignancy worldwide, accounting for one fifth of all cancer-related deaths (1). There are different trends of lung cancer incidence and mortality throughout Europe, mostly reflecting different phases of smoking epidemic in individual countries. In many European countries, the rates in men have recently decreased or stabilized, while the rates in women increased (2-4). Because the majority of lung cancer deaths are attributed to tobacco smoking, any decline or deceleration in the lung cancer death rates could be attributed to the past antismoking interventions (5,6). Early indicators of progress in tobacco-smoking control are lung cancer trends in young adults (6).

About 90% of lung cancers in men and 83% in women are caused by smoking (7). The risk of developing lung cancer is affected by the level of consumption and duration of smoking (8), as well as the level of exposure to environmental tobacco smoke (9). The second most important cause of lung cancer is radon, which was estimated to be responsible for 9% of lung cancer deaths in European countries (10). Other risk factors include exposure to asbestos (11), silica (12), nitrogen oxides (13), radiation to the chest as part of the treatment of malignant diseases (14-16), and scarring on the lungs due to tuberculosis or recurrent pneumonia (17).

Currently in Croatia, lung cancer is the most common cancer in men and the fifth most common cancer in women, accounting for more than 2000 and 600 deaths per year, respectively (18,19). The aim of this study was to provide an overview of the temporal trends of lung cancer incidence and mortality in Croatia for the period 1988-2008.

## Materials and methods

### Data sources

Incidence data for the period 1988-2008 were obtained from the Croatian National Cancer Registry. The Registry, founded in 1959, covers the whole Croatian population (approximately 4.4 million persons) and relies on mandatory cancer notifications from primary and secondary health care sources and death certificates from the Croatian Bureau of Statistics. The Registry has contributed data to the last three volumes of the Cancer Incidence in Five Continents series (20-22). In addition to incidence data, these publications report respective indices of data quality (proportion of morphologically verified cases, proportion of cases registered from death certificates only, and mortality to incidence ratio) (20-22). Lung cancer was defined as ICD-10 codes C33 and C34 and ICD-9 code 162. The number of lung cancer deaths was obtained from the World Health Organization (WHO) mortality database (19). Population estimates from Population Division of the Department of Economic and Social Affairs of the United Nations were used for calculating all incidence and mortality rates (23).

### Statistical analysis

Age-standardized rates of cancer incidence in Croatia and truncated age-standardized rates (for ages 30-64) were calculated by the direct standardization method, using the world standard population as a reference (24). To describe incidence and mortality time trends, we carried out joinpoint regression analysis using the software Joinpoint Regression Program, Version 3.5.2, October 2011. The analysis included logarithmic transformation of the rates, standard error, maximum number of five joinpoints, and minimum of four years between two joinpoints. All other program parameters were set to default values. The aim of the approach is to identify possible joinpoints where a significant change in the trend occurs. The method identifies joinpoints based on regression models with 0-5 joinpoints. The final model selected was the most parsimonious of these, with the estimated annual percent change (EAPC) based on the trend within each segment (25). To quantify the trend over the whole period, the average annual percent change (AAPC) was calculated. The AAPC is computed as a geometric weighted average of the EAPC trend analysis, with the weights equal to the lengths of each segment during the pre-specified fixed interval. If an AAPC lies entirely within a single joinpoint segment, the AAPC is equal to the EAPC for that segment. In these cases we reported the EAPCs (26).

In describing trends, the terms “significant increase” or “significant decrease” signify that the slope of the trend was statistically significant (*P* < 0.05). For non-statistically significant trends (*P* > 0.05), we used the terms “stable” (for EAPC between -0.5% and 0.5%), “non-statistically significant increase” (for EAPC>0.5%), and “non-statistically significant decrease” (for EAPC<-0.5%). All statistical tests were two sided.

## Results

### Men

**Figure 1:**
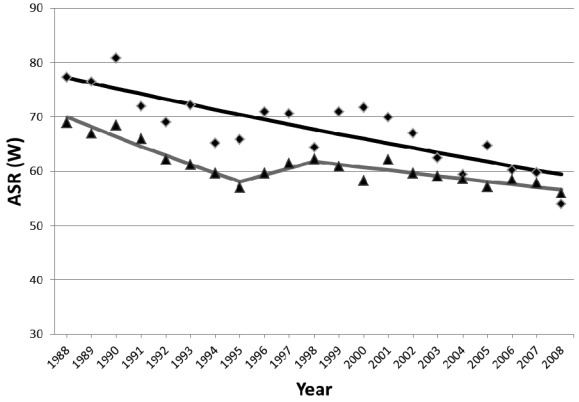
Joinpoint analyses of incidence (rhombs) and mortality (triangles) for lung cancer in Croatian men, 1988-2008. ASR (W) – age standardized rate (using world standard population).

The number of new lung cancer cases in men remained stable (Table 1). Crude incidence rates did not change and age-standardized rates (ASR) declined (Figure 1). Lung cancer age-standardized incidence rates in men decreased by one fifth, from 75.1/100 000 in the first five years (1988-1992) to 59.6/100 000 in the last five years (2004-2008). Mortality age-standardized rates in men changed from 66.5/100 000 in the first five years (1988-1992) to 57.6/100 000 in the last five years (2004-2008), with an overall percent change of -13.4%. Joinpoint analysis (Table 2) showed a significant decrease in the incidence, with EAPC of -1.3% (95% confidence interval [CI], -1.7% to -0.9%). For mortality, joinpoint analysis (Table 3) identified three trends. From 1988 to 1995, mortality significantly decreased, with EAPC of -2.6% (95% CI, -3.4% to -1.8%). From 1998 onwards, there was a non-significant increase of 2.1% (95% CI, -3.9% to 8.5%) followed by another decreasing trend of -0.9% (95% CI, -1.3% to -0.4%). AAPC was -1.1% (95% CI, -1.9% to -0.2%). Analysis by ten-year age groups revealed that both incidence and mortality rates decreased significantly for all age groups <70 years old during the study period (Tables 2 and 3).

**Table 1:** Lung cancer incidence and mortality in Croatia in the period 1988-2008. Number of cases, crude rate, and age standardized rate per 100 000

Year	Incidence			Mortality		
	N	crude rate	ASR*	N	crude rate	ASR
Men:						
1988	2115	97.4	77.3	1866	85.9	68.9
1989	2149	98.7	76.5	1848	84.9	67.0
1990	2307	105.6	80.7	1944	89.0	68.4
1991	2074	94.3	72.0	1900	86.4	66.0
1992	2051	92.5	69.0	1843	83.2	62.2
1993	2236	100.1	72.2	1880	84.2	61.2
1994	2080	92.6	65.2	1893	84.3	59.7
1995	2142	95.2	65.9	1848	82.1	57.1
1996	2343	104.4	71.0	1973	87.9	59.7
1997	2354	105.7	70.7	2050	92.0	61.4
1998	2167	98.2	64.4	2095	94.9	62.2
1999	2394	109.5	71.0	2070	94.7	60.9
2000	2457	113.2	71.7	2000	92.2	58.3
2001	2418	112.0	70.0	2161	100.1	62.1
2002	2351	109.3	67.0	2102	97.7	59.6
2003	2219	103.4	62.5	2110	98.3	59.1
2004	2152	100.5	59.5	2123	99.1	58.6
2005	2346	109.7	64.7	2086	97.6	57.2
2006	2181	102.2	60.2	2145	100.5	58.5
2007	2217	104.1	59.7	2170	101.9	57.8
2008	2024	95.2	53.9	2139	100.6	56.0
Women:						
1988	342	14.8	9.0	307	13.3	8.0
1989	383	16.5	9.7	347	15.0	8.6
1990	441	18.9	11.3	341	14.6	8.5
1991	400	17.0	10.0	350	14.9	8.8
1992	386	16.3	9.2	367	15.5	8.6
1993	389	16.2	9.0	319	13.3	7.3
1994	408	16.9	9.4	346	14.3	8.0
1995	410	17.0	9.1	342	14.1	7.6
1996	507	21.0	11.1	417	17.3	9.0
1997	480	20.0	10.3	401	16.7	8.5
1998	439	18.5	9.3	474	20.0	9.9
1999	525	22.3	11.2	425	18.1	8.8
2000	589	25.2	12.9	478	20.5	10.4
2001	542	23.3	11.7	455	19.6	9.6
2002	590	25.5	12.8	496	21.4	10.3
2003	555	24.0	11.8	530	22.9	10.9
2004	586	25.4	12.0	512	22.2	10.9
2005	654	28.4	13.5	554	24.1	11.0
2006	664	28.9	14.3	572	24.9	11.7
2007	569	24.8	11.7	583	25.4	11.8
2008	514	22.4	10.4	611	26.7	12.3

*ASR (W) – age standardized rate (using world standard population).

**Table 2:** Joinpoint analysis of age-specific and age-standardized rates of lung cancer incidence in Croatia (1988-2008)

Age (years)	Trend 1		Trend 2		Trend 3		AAPC (1988-2008)
	years	EAPC	years	EAPC	years	EAPC	
Men:							
30-39	1988-2008	- 6.3^†^					-6.3^†^
40-49	1988-2000	0.9	2000-2008	- 5.5^†^			-1.7^†^
50-59	1988-1995	- 4.7^†^	1995-2008	- 0.6			-2.0^†^
60-69	1988-2001	- 0.9^†^	2001-2008	- 3.8^†^			-2.0^†^
70-79	1988-1995	- 0.6	1995-2001	3.4	2001-2008	- 3.4*	-0.4
>80	1988-2008	0.1					0.1
Age standardized:							
overall	1988-2008	- 1.3^†^					-1.3^†^
truncated (30-64)	1988-2008	- 2.0^†^					-2.0^†^
Women:							
30-39	1988-2008	- 3.2^†^					-3.2
40-49	1988-2008	1.9^†^					1.9^†^
50-59	1988-2008	3.5^†^					3.5*
60-69	1988-2008	1.0^†^					1.0^†^
70-79	1988-2004	2.7^†^	2004-2008	- 6.8			0.7
>80	1988-2008	1.3^†^					1.3^†^
Age standardized							
overall	1988-2008	1.7^†^					1.7^†^
truncated (30-64)	1988-2008	2.2^†^					2.2^†^

*EAPC – estimated annual percent change; AAPC – average annual percent change.

†Statistically significant trend.

**Table 3:** Joinpoint analysis of age-specific and age-standardized rates of lung cancer mortality in Croatia (1988-2008)

Age (years)	Trend 1		Trend 2		Trend 3		AAPC (1988-2008)
	years	EAPC	Years	EAPC	years	EAPC	
Men:							
30-39	1988-2008	-8.7^†^					-8.7^†^
40-49	1988-2000	0.2	2000-2008	-4.3^†^			-1.6
50-59	1988-2000	-3.2^†^	2000-2008	1.2			-1.5^†^
60-69	1988-1993	-2.8^†^	1993-1999	0.8	1999-2008	-2.2^†^	-1.5^†^
70-79	1988-1995	-1.5	1995-2002	3.1^†^	2002-2008	-0.4	0.4
>80	1988-1995	-4.8	1995-2008	3.3^†^			0.4
Age standardized:							
overall	1988-1995	-2.6^†^	1995-1998	2.1	1998-2008	-0.9^†^	-1.1^†^
truncated (30-64)	1988-2008	-1.8^†^					-1.8^†^
Women:							
30-39	1988-2008	-1.2					-1.2
40-49	1988-2008	2.9^†^					2.9^†^
50-59	1988-2008	2.7^†^					2.7^†^
60-69	1988-2008	2.1^†^					2.1^†^
70-79	1988-2008	2.0^†^					2.0^†^
>80	1988-2008	2.2^†^					2.2^†^
Age standardized:							
overall	1988-1994	-1.0	1994-2008	3.1^†^			1.9^†^
truncated (30-64)	1988-2008	2.7^†^					2.7^†^

*EAPC – estimated annual percent change; AAPC – average annual percent change.

†Statistically significant trend.

### Women

**Figure 2:**
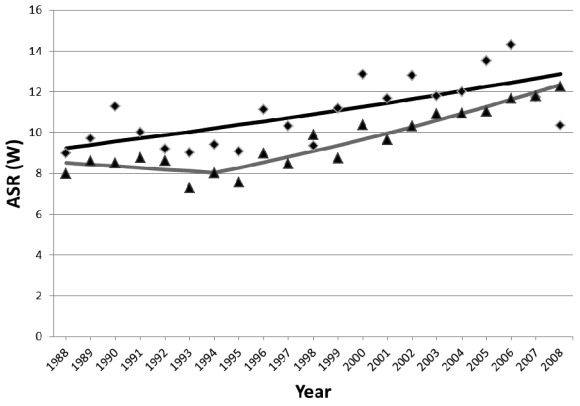
Joinpoint analyses of incidence (rhombs) and mortality (triangles) for lung cancer in Croatian women, 1988-2008. ASR (W) – age standardized rate (using world standard population).

In women, there was an increase in the number of cases and ASRs (Figure 2). Age-standardized incidence rates increased by 26.5%, from 9.8/100 000 in the first five-years (1988-1992) to 12.4/100 000 in the last five-years (2004-2008). Mortality age-standardized rates increased by 35.3%, from 8.5/100 000 in the first five years (1988-1992) to 12.5/100 000 in the last five years (2004-2008). Joinpoint analysis showed a significantly increasing trend of incidence, with EAPC of 1.7% (95% CI, 0.8% to 2.5%) (Table 2). Joinpoint analysis of mortality (Table 3) identified one joinpoint in 1994. The first trend showed non-significant decrease, with EAPC of -1.0% (95% CI, -4.1% to 2.2%), followed by a significant increase, with EAPC of 3.1% (95% CI, 2.3% to 3.9%) from 1994 to the end of the study period. AAPC was 1.9% (95% CI, 0.9% to 2.9%) throughout the whole period. Analysis by ten-year age groups showed that both incidence and mortality increased significantly in all age groups older than 40 years and decreased in younger women (30-39 years). (Tables 2 and 3)

## Discussion

This study confirmed the findings that, despite the overall decreasing trend, Croatia is still among the European countries with the highest male lung cancer incidence and mortality rate (27,28). With male ASR incidence and mortality of 59.6 and 57.6/100 000 men, respectively, it is in the top five of the 40 analyzed European countries. Hungary has the highest estimated male ASR of mortality, of 73.5/100 000, while Cyprus has the lowest, of 21.3/100 000. Incidence and mortality in women are considerably lower. Compared to other European countries, Croatia has an intermediate incidence and mortality rate (12.4 and 12.5/100 000, respectively). Denmark has the highest mortality rate of 30/100 000 and Belarus the lowest – 3.7/100 000 (28).

More than a quarter of all adult inhabitants of Croatia are every-day smokers (29). A survey conducted in 1972 showed a prevalence of daily cigarette smoking of 56.9% in men and 10.1% in women. In the period between 1972 and 1997, the prevalence decreased among men and increased among women. During the last decade, there has been a reduction in the frequency of smokers of both sexes (29,30). Our results show that lung cancer incidence and mortality rates in men have been constantly decreasing in most of the age groups. This trend is in agreement with the decrease in the prevalence of male smokers observed during the same period (29). On the other hand, women showed an increasing incidence trend, although their age standardized incidence rates are still 5 times lower than those of men. This increasing incidence trend follows the increase in the prevalence of female smokers (29) and reflects a lung cancer epidemic in women. Still, though not significant, we observed a stabilization and beginning of a decreasing trend in younger women (30-39 years). Together with the initial reduction in the frequency of smokers among women during the last decade (30), this might be an early indicator that lung cancer incidence and mortality in women will reach a plateau or start declining in the future. The trends in Croatia are similar to those in most European countries. The mortality trends in men are largely encouraging, since there are general declines in most European countries, particularly during the last two decades, while there are still increases in parts of Southern (Portugal and the Republic of Macedonia) and Eastern Europe (Bulgaria, Romania, and Moldova) (27). Among women, mortality rates have reached a plateau or are beginning to decline in a number of Eastern European countries (Hungary, Poland, and the Czech Republic), and in Northern Europe (Denmark, Iceland, and the United Kingdom), which reflects recent changes in smoking habits. The trends in Western Europe (France and the Netherlands) and Southern Europe (Spain) are increasing (27).

Croatia still bears a major smoking-related health burden. Some of the most obvious barriers to implementing successful smoking prevention program initiatives include very limited resources, recent transnational tobacco industry’s interest in the Croatian market, and the social acceptability of smoking. By ratifying the Framework Convention for Tobacco Control, Croatia did integrate European tobacco bans, but smoking is still allowed in most of the bars and cafes (31). Because of a large number of older Croatians who have been smoking for decades, the rise in the number of tobacco-related deaths is expected to continue (31). Such trends encourage the implementation of smoking prevention and cessation policies, particularly those targeting younger population, especially women.
